# Resistive Switching in Bigraphene/Diamane Nanostructures Formed on a La_3_Ga_5_SiO_14_ Substrate Using Electron Beam Irradiation

**DOI:** 10.3390/nano13222978

**Published:** 2023-11-20

**Authors:** Evgeny V. Emelin, Hak Dong Cho, Vitaly I. Korepanov, Liubov A. Varlamova, Darya O. Klimchuk, Sergey V. Erohin, Konstantin V. Larionov, Deuk Young Kim, Pavel B. Sorokin, Gennady N. Panin

**Affiliations:** 1 Institute of Microelectronics Technology and High-Purity Materials, Russian Academy of Sciences, 142432 Chernogolovka, Moscow Region, Russia; eemelin@iptm.ru (E.V.E.); korepanov@iptm.ru (V.I.K.); 2 Quantum-Functional Semiconductor Research Center, Dongguk University, Seoul 04620, Republic of Korea; hakcho1@dongguk.edu (H.D.C.);; 3 Laboratory of Digital Material Science, National University of Science and Technology MISIS, 119049 Moscow, Russia; varlamova.la@misis.ru (L.A.V.); erohin.sv@misis.ru (S.V.E.);; 4 Physical Chemistry Department, National University of Science and Technology MISIS, 119049 Moscow, Russia; 5 Department of Semiconductors and Dielectrics, National University of Science and Technology MISIS, 119049 Moscow, Russia; 6 Division of Physics and Semiconductor Science, Dongguk University, Seoul 04620, Republic of Korea

**Keywords:** graphene, diamane, resistive switching, memristor, electron beam irradiation

## Abstract

Memristors, resistive switching memory devices, play a crucial role in the energy-efficient implementation of artificial intelligence. This study investigates resistive switching behavior in a lateral 2D composite structure composed of bilayer graphene and 2D diamond (diamane) nanostructures formed using electron beam irradiation. The resulting bigraphene/diamane structure exhibits nonlinear charge carrier transport behavior and a significant increase in resistance. It is shown that the resistive switching of the nanostructure is well controlled using bias voltage. The impact of an electrical field on the bonding of diamane-stabilizing functional groups is investigated. By subjecting the lateral bigraphene/diamane/bigraphene nanostructure to a sufficiently strong electric field, the migration of hydrogen ions and/or oxygen-related groups located on one or both sides of the nanostructure can occur. This process leads to the disruption of sp^3^ carbon bonds, restoring the high conductivity of bigraphene.

## 1. Introduction

Memristors as resistive switching memory devices play a critical role in the energy-efficient implementation of artificial intelligence. Memristors possess unique properties that stem from their ability to operate at atomic scales, allowing, for example, the formation or rupture of metallic filaments using voltage signals to encode synaptic weights [1,2]. The high endurance and sub-nanoscale switching speeds of memristors contribute to their low energy consumption, making them suitable for efficient computing. Furthermore, the memristor’s resistive switching performance is influenced by its previous states, allowing for adaptive behavior and learning capabilities in neuromorphic systems. This property enables the development of artificial intelligence systems that can mimic the synaptic plasticity observed in biological neural networks [3].

Two-dimensional materials represent a novel class of nanostructured materials that hold great promise as emerging materials for ultra-thin semiconductor devices in the future [4]. This family of materials encompasses a wide range of options, including metals and insulators, offering a rich collection of nanostructured 2D materials that can be utilized for designing diverse devices [5,6,7]. In the present era, 2D materials have gained significant attention due to their unique properties, which arise from their low-dimensional nature and distinctive physical and chemical characteristics [8].

Graphene-based layer heterostructures, renowned for their remarkable electrical, thermal, and mechanical properties, exhibit immense potential for memristor applications. Recent studies have showcased the promise of graphene/graphene oxide heterostructures in resistive memories [9,10]. In vertical configurations, resistive switching has been observed in graphene oxide structures with on/off ratios of approximately 20 [11] and 100 [12] for Cu-Pt and Al-Al electrodes, respectively. The mechanism behind this switching involves the migration of metal atoms and/or a redox process. In planar structures on Si/SiO_2_ substrates, conductive graphene filaments within a graphene oxide matrix mainly govern the switching behavior. By manipulating the sp^2^/sp^3^ domains using oxidation/reduction processes, the desired graphene/graphene oxide-based memories can be achieved.

Ultra-thin diamond film, also known as diamane [13], emerges as another promising candidate for memristor applications. Initially regarded as a purely theoretical concept, diamane has been successfully observed, marking a significant milestone [14,15,16,17]. The band structures of diamane exhibit semiconducting behavior and possess a wide direct band gap, which renders it highly promising across various scientific fields [13]. The stability of diamane significantly deviates from that of diamond due to specific thermodynamic conditions. A pristine surface of bilayer diamane is inherently unstable; however, this instability can be reversed using the deposition of external atoms on bilayer graphene [13]. As a result, the bilayer graphene layers connect, leading to the formation of robust sp^3^-hybridized bonds. This chemically induced phase transition facilitates the controlled formation of high-resistivity diamane islands within the bilayer graphene structure by selectively attaching reference atoms. This controlled formation process allows for the precise engineering of the electrical properties of the composite system.

In recent research [18], the formation of diamondized regions in polymethyl methacrylate (PMMA)-covered bilayer graphene on the La_3_Ga_5_SiO_14_ surface has been successfully demonstrated. In the investigation, two layers of graphene were transferred onto a langasite substrate and subjected to focused electron beam irradiation through a layer of polymethyl methacrylate. The results of these studies revealed the local diamondization of bilayer graphene during the irradiation process. The observed results can be explained using the framework of the theory of a chemically induced phase transition, which is associated with the formation of sp^3^ bonds between carbon atoms and hydrogen and oxygen atoms released from PMMA and langasite, respectively, upon irradiation of the structure with an electron beam. Theoretical calculations and experimental evaluations of the sp^3^-hybridized carbons in the modified bilayer graphene structure on langasite provide evidence for the formation of diamane in the irradiated regions. These findings contribute to our understanding of the mechanisms underlying the transformation of bilayer graphene into diamane and pave the way for further exploration of the properties and potential applications of this novel material.

The focus of this study is on the resistive switching phenomenon in this lateral two-dimensional composite structure. The first part of this paper delves into an examination of the current–voltage characteristics of bilayer graphene, revealing differences in behavior between the initial and electron beam-irradiated structures. The second part of this study involves the analysis of the structural features of the formed nanomaterial. This analysis was conducted through the mapping of the intensity ratio between the D and G Raman bands. The final part of the work is dedicated to the study of the resistive switching effect in the formed bilayer graphene/diamane/bilayer graphene structure in an external electric field and the theoretical investigation of the response of the structure to the electric field.

## 2. Methods

### 2.1. Experimental Methods

Graphene monolayers were synthesized using chemical vapor deposition (CVD) on a copper catalyst foil. The CVD process was carried out in a horizontal quartz tube measuring 2400 mm in length and 152 mm in diameter, which was placed inside a six-zone furnace with a length of 1500 mm. A 25 μm thick copper foil, specifically a 10 × 30 cm^2^ foil with a purity of 99.999% from Alfa Aesar, was loaded into the CVD reactor and evacuated to a base vacuum pressure of less than 10^−4^ Torr.

To initiate the growth of graphene layers, the temperature inside the chamber was raised to 1020 °C. A mixture of methane (CH_4_) and hydrogen (H_2_), along with argon (Ar) as the carrier gas, was introduced into the reaction chamber at a pressure of 600 mTorr. The flow rates of the gases were set at 40 cm^3^/min for the CH_4_, 100 cm^3^/min for the H_2_, and 2000 cm^3^/min for the Ar. The growth process was carried out for 30 min, after which the reaction chamber was cooled down to room temperature at an average rate of 14 °C/min, maintaining the same flow rates of Ar and H_2_, but without the presence of methane.

To transfer the grown graphene monolayers, a common two-step process was employed [19] (see Figure S1 in the Supplementary Materials for more detail). First, the graphene layers on the copper foil were removed from the reverse side using oxygen plasma with a power of 60 W. Then, the remaining graphene layers were transferred onto the polished surface of a La_3_Ga_5_SiO_14_ (LGS) substrate. The transfer was facilitated by using a poly(methyl methacrylate) (PMMA) layer as support. The PMMA layer was spin-coated onto the graphene-covered copper foil and dried in an oven at 120 °C for 10 min. Subsequently, the PMMA/graphene layer was immersed in a solution of distilled water to facilitate the separation of the graphene layer from the copper foil. The PMMA was then removed by soaking the sample in acetone or isopropyl alcohol, and the resulting single-layer graphene (SLG) on the LGS substrate was rinsed with a 30% hydrochloric acid (HCl) solution at 60 °C for 30 min to eliminate any residual Fe^3+^ ions. This process ensured the preparation of a high-quality graphene layer with a low defect density on the LGS substrate.

The same procedure was repeated to transfer the upper layer of graphene onto the previously transferred graphene layer. Before the transfer procedure, the surface of the lower layer was thoroughly cleaned with solvents and an ultrasonic bath to ensure a tight fit between the graphene monolayers.

The graphene layers on the substrates were analyzed using Raman scattering with a CRM 200 spectrometer (WiTec, Ulm, Germany). A 100× objective (Olympus, NA 0.9, Rochester, NY, USA) was used, along with a 532 nm (or 488 nm) laser with a power of 1 mW (or 2.5 mW, 50 mW). A GX polarized filter-AN360 (Olympus) was also employed. Each spectrum was obtained by taking 10 measurements within a 10 s accumulation time. Multiple analyses, ranging from 3 to 6, were performed at different locations for each sample.

To prepare the samples for further analysis, a layer of PMMA-950 resist with a thickness of 300 nm was deposited onto the system. These samples were irradiated using an EVO-50 scanning electron microscope equipped with a Nanomaker electron beam control. The Raman spectra of nanostructures obtained using irradiation with a focused electron beam were measured using a Bruker Senterra micro-Raman system. The excitation wavelength for the Raman measurements was 532 nm, and the laser power at the sample point was set to 10 mW. Each point on the map was captured for 2 × 20 s. Transport measurements of the nanostructures were conducted using a microprobe station EPS150TRIAX (Microtron, Eindhoven, The Netherlands) and a Keithley 2636B System SourceMeter^®^ SMU Instrument (Tektronix, Beaverton, OR, USA).

### 2.2. Computational Details

The effect of a lateral electric field on the atomic structure of graphene/diamane/graphene was investigated using density functional theory (DFT) [20,21]. The calculations were performed within the generalized gradient approximation using the Perdew–Burke–Ernzerhof (PBE) parametrization [22]. The SIESTA code [23] was employed using the double-ζ plus polarization (DZP) basis set of numerical atomic orbitals [24]. To account for core electrons, norm-conserving Troullier–Martins pseudopotentials [25] in their fully nonlocal representation [26] were used. The real space mesh cutoff was set to 200 Ry. Periodic boundary conditions were applied along the nanoribbon direction, while a vacuum region of 15 Å was introduced in the perpendicular direction to the ribbon edge. The Brillouin zone was sampled using a 16 × 1 × 1 Monkhorst–Pack grid [27]. The atomic structure was relaxed until the maximum interatomic force fell below 0.04 eV/Å. The ribbon edges were passivated with hydrogen atoms. To simulate the external electric field, a periodic sawtooth-type potential was applied perpendicular to the ribbon edge, taking into account the slab dipole correction to the electrostatic potential and energies [28].

## 3. Results and Discussion

Recently, the formation of diamane nanostructures in bilayer graphene on a La_3_Ga_5_SiO_14_ substrate under focused electron beam irradiation was reported [18]. The observed results can be explained using the framework of the theory of a chemically induced phase transition from bilayer graphene to diamane [13]. This transition is associated with the release of hydrogen and oxygen atoms from PMMA and langasite, respectively, due to the “knock-on” effect caused by electron beam irradiation. A similar outcome was observed in the formation of diamond nanoclusters within the carbon network. In this case, hydrogen atoms displaced from the dodecyl groups due to the “knock-on” effect penetrate the layered carbon structure and subsequently form diamond clusters with sizes reaching up to 10 nm [29]. The modified structure of bilayer graphene on langasite was theoretically calculated, and the experimental evaluation of the fraction of sp^3^-hybridized carbon confirmed the formation of diamane nanoclusters in the irradiated regions of bilayer graphene.

**Figure 1:**
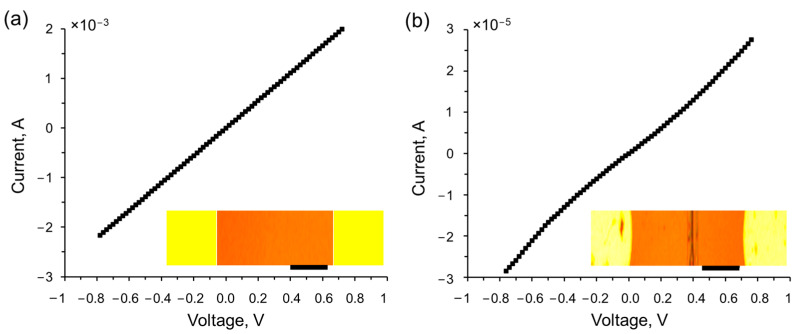
Current–voltage characteristics of a two-layer graphene with Al/Cr side electrodes on a La_3_Ga_5_SiO_14_ substrate, both prior to the electron beam-induced transition from bilayer graphene to diamane (**a**) and following the localized phase transition (**b**). The insets provide optical images of the structure, with the two-layer graphene represented in brown and the two Al/Cr electrodes highlighted in yellow. The region of the bigraphene that has undergone local irradiation with an electron beam using PMMA to convert it to diamane is shown as a vertical dark stripe. The scale bar in the images corresponds to 1 µm.

Figure 1 showcases the current–voltage characteristics of a bilayer graphene, which was transferred onto a La_3_Ga_5_SiO_14_ substrate with Al/Cr side electrodes, both before (a) and after (b) the electron beam-induced transition from bilayer graphene to diamane. The insets in Figure 1 provide optical images of the structure: the unirradiated bilayer graphene on a La_3_Ga_5_SiO_14_ substrate with two Al/Cr electrodes (a), and the irradiated structure with an electron beam passing through PMMA along a specific line to locally convert bilayer graphene to diamane (b). Initially, the bilayer graphene on a La_3_Ga_5_SiO_14_ substrate with Al/Cr side electrodes exhibits a linear current–voltage characteristic and a resistance of 360 Ohms (Figure 1a). However, upon irradiation with a focused electron beam, a diamane structure is formed, characterized by nonlinear charge carrier transport behavior and a significant increase in resistance up to 35 kΩ (Figure 1b).

**Figure 2:**
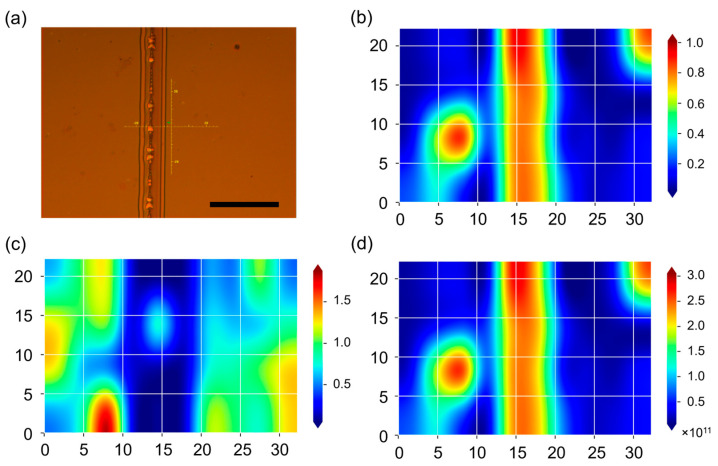
(**a**) Optical image of the surface of two-layer graphene on a La_3_Ga_5_SiO_14_ substrate with PMMA irradiated using an electron beam (vertical stripe) at an accelerating voltage of 25 kV and a dose of 1 mC/cm^2^. The scale bar in the image corresponds to 1 µm. Bubble-shaped areas formed indicate the release of excess hydrogen from irradiated PMMA. Intensity ratio map of the D and G (**b**) and the 2D and G (**c**) Raman bands of two-layer graphene after local electron irradiation in the form of a vertical stripe. (**d**) sp^3^ defect density distribution (cm^−2^) estimated from the D peak.

In Figure 2b, mapping of the intensity ratio between the D and G Raman bands of a two-layer graphene system is presented. This mapping was performed in a region of approximately 27 × 24 µm surrounding a vertical stripe, which was obtained using irradiation with a focused electron beam, as depicted in Figure 2a. Previous studies [18] have demonstrated significant modifications in the Raman modes within the irradiated regions of bilayer graphene. Specifically, irradiation with an electron beam results in a notable increase in the intensity of the D peak, indicating an elevated density of sp^3^-hybridized carbon. The Raman mode at approximately 1345 cm^−1^ corresponds to defects responsible for the formation of sp^3^-hybridized regions, a characteristic commonly observed in graphene oxide (GO). Previous research has shown that electron beam irradiation of GO effectively reduces it, leading to a decrease in the density of sp^3^-hybridized carbon and a relative decrease in the intensity of the D peak without any shift. In the case of a similar bilayer graphene structure, irradiation with an electron beam resulted in a shift of the D peak to 1335 cm^−1^ and an increase in intensity [18]. Additionally, a peak at 1319–1337 cm^−1^, indicative of diamond-like hybridization [30], was observed in few-layer graphene, while the D peak in graphene is typically located at approximately 1350 cm^−1^ [31]. Consequently, the concentration of sp^3^-hybridized carbon increases, and the characteristic peak shifts toward the Raman mode associated with diamane. The Raman spectra obtained from non-irradiated and irradiated areas of bilayer graphene are shown in Figure S2 of the Supplementary Materials.

The content of sp^3^ carbon in bigraphene irradiated with a locally focused electron beam can be obtained from the approach in [32,33] using the equation $n_{D}\left(cm^{-2}\right)=\frac{10^{14}}{\pi^{2}(C_{A}\left(r_{A}^{2}-r_{S}^{2}\right)+C_{S}r_{S}^{2}}\frac{I\left(x\right)}{I\left(G\right)}$ proposed in [32]. For the D peak, *C_A_* = 4.2, *C_S_* = 0, *r_A_* = 3 nm, and *r_S_* = 1 nm, and for the D’ peak, *C_A_* = 0.5, *C_S_* = 0.33, *r_A_* = 2.6 nm, and *r_S_* = 1.4 nm [18].

If we assume that defects of the “vacancy” type can be disregarded, the concentration of sp^3^ carbon can be estimated using the intensity ratio *I*(D)/*I*(G), as depicted in Figure 2b,d. Our calculations indicate that the fraction of sp^3^ carbon in the irradiated region amounts to 10^12^ cm^–2^ (Figure 2d) [18].

**Figure 3:**
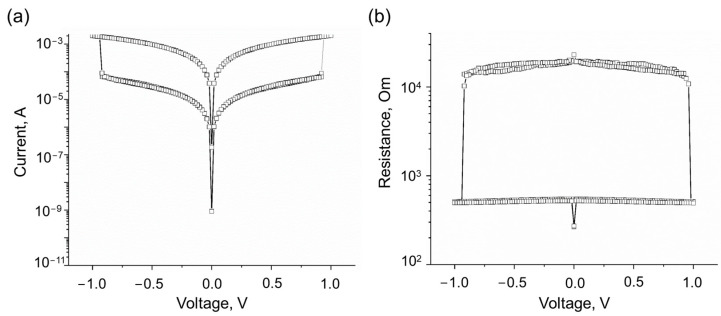
Current–voltage characteristics of an Al/Cr/bigraphene/diamane/bigraphene/Al/Cr nanostructure on a La_3_Ga_5_SiO_14_ substrate formed by electron beam irradiation of bigraphene demonstrating resistive switching (**a**) and changes in its resistance (**b**) during a bias voltage sweep.

Figure 3 illustrates the current–voltage characteristics of a nanostructure consisting of bilayer graphene, diamane, and bilayer graphene, with Al/Cr electrodes on a La_3_Ga_5_SiO_14_ substrate (a). Additionally, Figure 3b showcases the changes in resistance during a bias voltage sweep.

The voltage sweeps from 0 V to −1 V and then to 1 V and back to 0 V resulting in resistive switching at −0.9 and 0.9 V from a high-resistance state (~20 kOm) to a low-resistance state (~0.5 kOm) and back, respectively, demonstrating an on/off ratio of ~40, which is comparable to previously reported characteristics of memristors and photomemristors based on 2D materials [9,11,12,34]. It should be noted that direct “writing” diamane memristor structures with bilayer graphene electrodes by using electron beam irradiation opens new possibilities for fabricating memristor devices on bilayer graphene built-in integral circuits using electron beam processing compatible with CMOS technology.

When graphene layers are exposed to the adsorption of atoms or molecular groups, they can bind together and undergo a chemically induced phase transition, resulting in the formation of a diamond film. It is important to note that this phase transition occurs when the adsorbing atoms or molecular groups have a radius comparable to that of a carbon atom. If the adsorbing species are too large, the steric factor hinders the complete coverage of the film surface. In this context, oxygen-containing groups of moderate size are particularly suitable for providing full passivation of the diamane film. These groups can effectively bind to the surface of the film, ensuring its stability and preventing the formation of defects. Furthermore, when a sufficiently strong electric field is applied to a lateral bigraphene/diamane/bigraphene nanostructure, it can induce the migration of hydrogen ions (and/or oxygen-related groups) that are located on one or both sides of the nanostructure. This migration process can break the sp^3^ carbon bonds and subsequently transform the structure into bigraphene. This alteration in the carbon bonding configuration can have significant implications for the electronic and structural properties of the nanostructure.

In the previous work [18], in which the formation of diamondized regions in PMMA-covered bilayer graphene on the La_3_Ga_5_SiO_14_ surface was demonstrated, we designed a corresponding model of a diamane film arranged on a substrate functionalized with hydrogen atoms from the outer side. During the relaxation process, the surface oxygen atoms of langasite shifted and formed connections with the carbon atoms at the interface, thereby stabilizing the geometry of the diamane film. The resulting structure exhibited a hexagonal diamane film with the $\left(10\overset{-}{1}0\right)$ surface, which displayed high stability. This finding supports the experimental suggestion of the diamondization of bilayer graphene through the treatment with H and O atoms.

**Figure 4:**
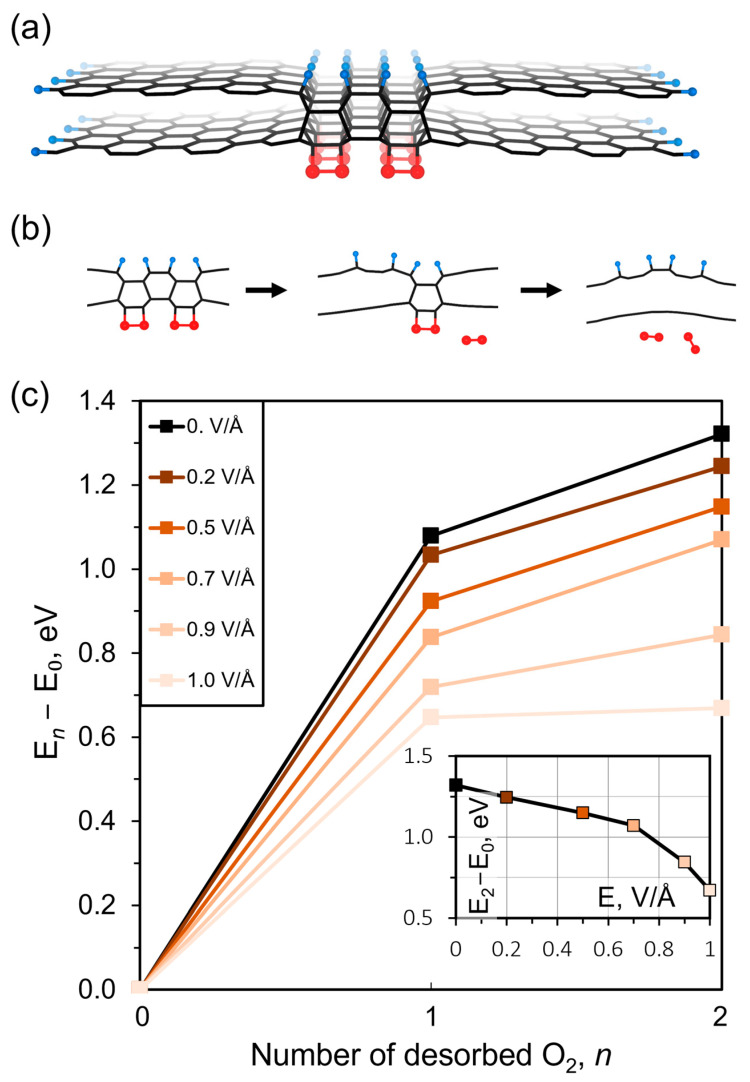
(**a**) A bilayer graphene ribbon with a diamond region passivated by hydrogen atoms on one side (blue balls) and peroxide groups on the other side (red balls). (**b**) The stages of oxygen atom detachment: the initial structure, detachment of the first oxygen molecule, and detachment of the second oxygen molecule. (**c**) The dependence of the diamond cleavage barrier, calculated as the difference between the initial stage and the stage with detached O_2_, for the desorption of one and two oxygen molecules from the diamond surface. The graph shows the variation in the cleavage barrier with different voltages of an applied electrical field. The inset provides a closer look at the dependence of the cleavage barrier for the desorption of two oxygen molecules on the applied electrical field.

In this study, we specifically examined the response of the 2D diamond structure to an external electric field. To simplify the model, we removed the La_3_Ga_5_SiO_14_ substrate and retained only oxygen atoms in the form of peroxide groups on one side, while hydrogen atoms released from the PMMA coating were present on the other side. This simplified model consisted of a bilayer graphene/diamond heterostructure that was periodic only along the *x*-axis, forming a ribbon-like structure, as depicted in Figure 4a.

The stability of the diamond film is closely related to the strength of the C-O and C-H bonds. Functional groups on the film surface contribute to its stability, and their desorption leads to the cleavage of the film. We defined the cleavage barrier of the diamond cluster as the energy difference between the original structure (*E*_0_) and the structure with one (*E*_1_) or two (*E*_2_) detached oxygen molecules.

Our findings indicate that a diamond-like nanoribbon with a minimum width of 1 nm (consisting of four atomic layers arranged perpendicular to the surface) is thermodynamically stable. Narrower ribbons are unstable and delaminate into bilayer graphene. As the ribbon width increases, the energy barrier for diamond cluster cleavage also increases (from 0.66 eV/O_2_ to 2.38 eV/O_2_). However, the response to an electric field remains the same for ribbons of different widths, allowing us to focus on studying the minimal structure.

The process of the sequential disruption of the diamond cluster using the displacement and desorption of peroxide groups is illustrated in Figure 4b. The energy barrier for cleavage is shown to decrease with the increasing magnitude of the applied electric field, as depicted in Figure 4c. In the absence of an electric field, the cleavage barrier due to oxygen desorption is relatively high. However, even a small electric field of 0.2 eV/Å reduces the barrier, and a field of 1.0 eV/Å decreases it by almost half. This significant response of C-O bonds to the electric field is attributed to their high polarity. On the other hand, the effect of the field on C-H bonds is negligible. Consequently, the desorption of oxygen atoms from the surface leads to the complete destruction of the diamane structure and the restoration of metallic conductivity in the system. Based on these results, we can conclude that effective control over the conductive properties of the synthesized material can be achieved by applying a bias voltage.

## 4. Conclusions

In this study, we investigated the resistive switching behavior of a lateral 2D composite structure consisting of bilayer graphene and diamane (2D diamond). The local diamondization of bilayer graphene on a La_3_Ga_5_SiO_14_ substrate under focused electron beam irradiation was observed. Raman spectroscopy analysis revealed an elevated density of sp^3^-hybridized carbon in the irradiated regions. The current–voltage characteristics of the bilayer graphene before and after the electron beam-induced transition demonstrated a significant increase in resistance upon the formation of the diamane structure.

Furthermore, the resistive switching behavior of a nanostructure consisting of bilayer graphene, diamane, and bilayer graphene was investigated. A voltage sweep from 0 V to −1 V and then to 1 V and back to 0 V resulted in a switch from a high-resistance state to a low-resistance state and back. This resistive switching behavior was attributed to the migration of oxygen-related groups, leading to the restoration of sp^2^ carbon bonds in the bilayer graphene.

In our theoretical investigation, we focused on understanding the influence of an electric field on the bonding of functional groups to the surface and the overall stability of a diamond film. To achieve this, we designed a graphene/diamane heterostructure, in which the diamond layer is stabilized by oxygen atoms in the form of peroxide groups from the langasite substrate on one side and hydrogen atoms released from the PMMA coating on the other side. This model represents a diamond nanoribbon embedded within a graphene bilayer. The stability of the diamond film is closely tied to the strength of the C-O and C-H bonds. The presence of functional groups on the film surface contributes to its stability, and their desorption can lead to the cleavage of the film. Our simulations demonstrate that when a sufficiently strong electric field is applied to a lateral bigraphene/diamane/bigraphene nanostructure, it can induce the migration of oxygen-related groups. This migration process results in the breaking of interlayer sp^3^ carbon bonds and the disruption of the diamane structure. The alteration in the carbon bonding configuration and the disruption of the diamane structure play a crucial role in this process, enabling the system to exhibit distinct electrical properties and undergo reversible transitions between high and low resistance states.

The results of this study highlight the potential of using bilayer graphene/diamane structures for resistive switching applications. The electron beam-assisted chemically induced phase transition of bilayer graphene to diamane provides a new technology for the direct ‘’writing’’ of memristor devices on bilayer graphene using electron beam lithography. It opens new possibilities for using large-scale bilayer graphene substrates for 2D layered memristor fabrication using CMOS-compatible technology. The ability to control the conductive properties of the formed structure using local phase transitions through bias voltage opens up possibilities for the development of novel memristor devices with improved performance. Further research can optimize of the fabrication process to integrate these memristor structures into CMOS devices for various applications, including the power-efficient implementation of artificial intelligence and advanced computing systems.

## Data Availability

No new data were created or analyzed in this study. Data sharing is not applicable to this article.

## Supplementary Materials

The following supporting information can be downloaded at: https://www.mdpi.com/article/10.3390/nano13222978/s1. Figure S1. The transfer of graphene layers on a substrate. Figure S2. The Raman spectra obtained from non-irradiated and irradiated areas of the bilayer graphene.
