# Supplementary material for: Resistive Switching in Bigraphene/Diamane Nanostructures Formed on a La3Ga5SiO14 Substrate Using Electron Beam Irradiation
Source: Nanomaterials (Basel). 2023 Nov 20;13(22):2978. doi: 10.3390/nano13222978 (PMC10674167; doi:10.3390/nano13222978)
Supplement: Supplementary file 1 [file nanomaterials-13-02978-s001.zip › nanomaterials-2698931-supplementary.pdf]

Supplementary Information

Resistive Switching in Bigraphene/Diamane Nanostructures Formed on a $La_3Ga_5SiO_{14}$ Substrate by Electron Beam Irradiation

Eugenii V. Emelin [1,†], Hak Dong Cho [2,†], Vitaly I. Korepanov [1], Liubov A. Varlamova [3], Darya O. Klimchuk [3,4], Sergey V. Erohin [3,5], Konstantin V. Larionov [3], Deuk Young Kim [2,6], Pavel B. Sorokin [3,5*] and Gennady N. Panin [1*]

[1] Institute of Microelectronics Technology and High-Purity Materials, Russian Academy of Sciences, Chernogolovka, 142432 Moscow region, Russia
[2] Quantum-Functional Semiconductor Research Center, Dongguk University, Seoul 04620, Republic of Korea
[3] Laboratory of Digital Material Science, National University of Science and Technology MISIS, 119049 Moscow, Russia
[4] Physical Chemistry Department, National University of Science and Technology MISIS, 119049 Moscow, Russia
[5] Department of Semiconductors and Dielectrics, National University of Science and Technology MISIS, 119049 Moscow, Russia
[6] Division of Physics and Semiconductor Science, Dongguk University, Seoul 04620, Republic of Korea

[*] Correspondence: pbsorokin@misis.ru (P.B.S.); panin@iptm.ru (G.N.P.)
[†] These authors contributed equally to this work.

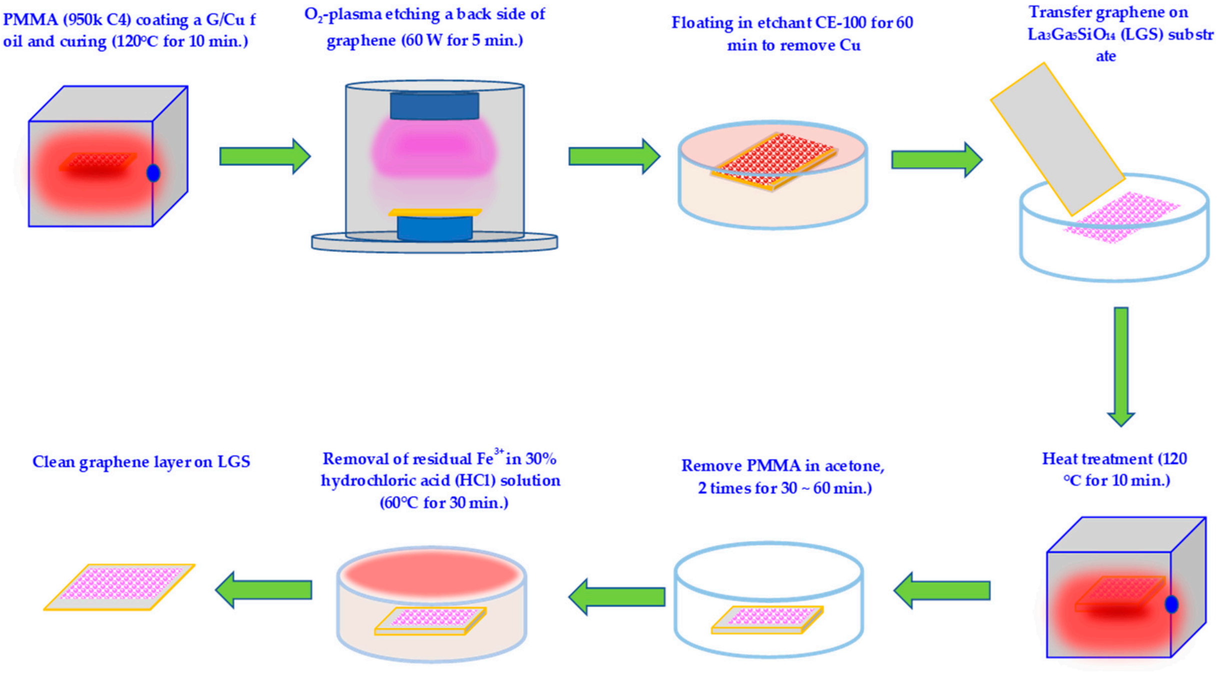

Figure S1. The transfer of graphene layers on a substrate.

To transfer the LPCVD grown graphene monolayers, a two-step process was employed (Fig. S1). First, the graphene layers on the copper foil were removed from the reverse side using oxygen plasma with a power of 60 W. Then, the remaining graphene layers were transferred onto a polished surface of a $La_3Ga_5SiO_{14}$ (LGS) substrate. The transfer was facilitated by using a poly(methyl methacrylate) (PMMA) layer as a support. The PMMA layer was spin-coated onto the graphene-covered copper foil and dried in an oven at 120 °C for 10 minutes. Subsequently, the PMMA/graphene layer was immersed in a solution of distilled water to facilitate the separation of the graphene layer from the copper foil. The PMMA was then removed by soaking the sample in acetone or isopropyl alcohol, and the resulting single-layer graphene (SLG) on the LGS substrate was rinsed with a 30% hydrochloric acid (HCl) solution at 60°C for 30 minutes to eliminate any residual Fe^{3+} ions. This process ensured the preparation of a high-quality graphene layer with a low defect density on the LGS substrate.

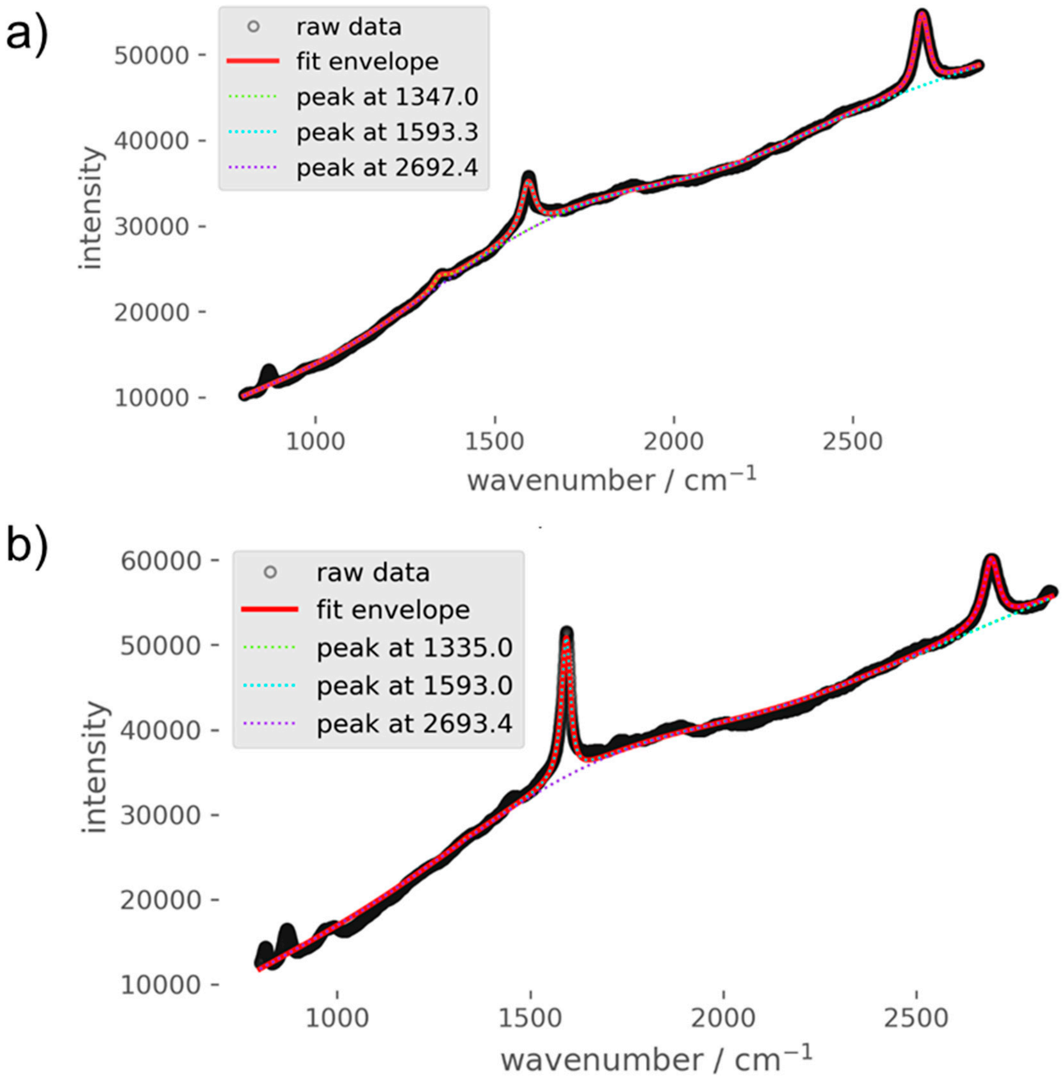

Figure S2. The Raman Spectra obtained from non-irradiated (a) and irradiated (b) areas of the bilayer graphene. The peaks at 1347.0, 1593.3 and 2692.4 cm^{-1} are clearly detected and fitted. Intensity ratio map of the D and G Raman bands of two-layer graphene after local electron irradiation in the form of a vertical stripe presented on Fig. 2b with sp^3 defect density distribution estimated from the D peak (Fig. 2d).
